# Exploring Hungarian teachers’ perceptions of inclusive education of SEN students

**DOI:** 10.1016/j.heliyon.2020.e03851

**Published:** 2020-05-08

**Authors:** Andrea Magyar, Anita Krausz, Ildikó Dóra Kapás, Anita Habók

**Affiliations:** a Szeged Center for Research on Learning and Instruction, University of Szeged, Szeged, Hungary; b Doctoral School of Education, University of Szeged, Szeged, Hungary; c University of Szeged, Szeged, Hungary; d Institute of Education, University of Szeged, Szeged, Hungary

**Keywords:** Education, Inclusive education, Special educational needs (SEN), SEN teachers, Teaching method, ICT, School-related factors, Hypothesized model of teachers' perceptions

## Abstract

The purpose of the current study is to identify Hungarian teachers' perceptions of special educational needs (SEN) and to explore their teaching practices in relation to ICT tools involved in classes with both SEN and typically developing students. A hypothesized model was developed and tested based on the relevant literature to map the relations of the variables. The research was conducted among primary and secondary school teachers (N = 121) using an adapted version of the *Audit of provision for students with special educational needs* self-evaluation scale (Stack, 2007), which was rounded out with ICT issues and background data on teachers' knowledge of SEN practices acquired at university. A path analysis showed significant links between the teaching- and learning-related factors on the scale. We also observed significant relationships between ICT variables, but the effect between the two factors is rather limited. The quality of curriculum provision is the only significant factor which has a significant effect on using ICT teaching material. Our model also highlighted that teachers’ knowledge of SEN acquired at university represents a determining factor in using ICT tools in SEN education. Teacher trainees who acquire comprehensive knowledge in their university education courses on teaching SEN students possess more profound skills to deal with them and possess higher-level competences in using ICT in their teaching practice. This study demonstrates that teaching SEN students in an integrated education system poses unique challenges; therefore, it is a relevant topic and an issue to be addressed. Our hope is that the results from this study will be useful in the future for schools and teachers educating students with special educational needs and that the findings will improve the overall understanding of inclusive education among schools and teachers.

## 1 Introduction

There is an increasing number of students in school systems worldwide who are diagnosed with special educational needs (SEN) (European Commission, 2017). In order to be able to perceive individual differences between students and respond to their particular needs, schools must be constantly improved. Employing appropriate approaches and teaching principles can also involve changes to school systems. Inclusive education is a way to address the learning needs of all children with a specific focus on those who are marginalised, excluded or characterised by any kind of special educational need. In this sense, *inclusion* is defined as “a process of addressing and responding to the diversity of needs of all children, youth and adults through increasing participation in learning, cultures and communities, and reducing and eliminating exclusion within and from education” (UNESCO, 2003, p. 7).

Researchers have collected a large amount of information on the cognitive benefits of inclusive education. Numerous studies have shown that inclusive education is advantageous to students' cognitive, social and affective development. Vidákovich and Habók (2019) focused on SEN students' cognitive development in online environment. De Leeuw et al. (2018) investigated the benefits of social inclusion for SEN students. Ahsan and Sharma (2018), Mulholland and Cumming (2016) and Saloviita (2020) explored the effect of inclusive education on the students’ affective development.

However, educational policies on inclusion vary from country to country (Ahsan and Sharma, 2018; Takala and Ahl, 2018). Local policy has a decisive impact; however, the broad category of SEN carries numerous legal benefits (UNESCO, 2003). Furthermore, the qualifications of each school's staff may also differ, since a number of educational institutions employ specialists, such as psychologists, social workers, trained counsellors, and educational or special educational assistants, to support teachers' work. The necessity of specifically trained staff depends on the level and type of each school. The teacher training curriculum also plays a crucial role. For example, teacher trainees in Spain enjoy the opportunity to take courses in special education for an additional year so that they may become competent in developing SEN students properly and professionally (Lasagabaster and de Zarobe, 2010). One of the essential objectives of teacher education should be to prepare practitioners adequately for upcoming challenges with the appropriate knowledge and methods needed to teach disabled students and those with diverse abilities.

Another important issue that may be beneficial in the development of SEN children is the use of information and communication technologies (ICT) in the teaching and learning process (Palomino, 2017). New methods can be introduced and education can be tailored to students’ needs through technology (Hardman, 2019; Yeni and Gecu-Parmaksiz, 2016). Another benefit of ICT is that providing a meaningful learning environment can support the development of higher order skills in SEN students (Csapó and Molnár, 2019; Gellerstedt et al., 2018; Mohamed, 2018; Vidákovich and Habók, 2019).

In Hungary, the education system operates differently. In most cases, there is no compulsory course for teacher trainees. However, at universities where pre-service teachers can attend compulsory SEN courses, their teaching methods are improved and they use ICT tools at a higher standard. The major aim of our study is to survey Hungarian teachers' perceptions of their schools' practices as regards SEN students and to explore how ICT is involved in their teaching process in an inclusive classroom. To shed light on these, we explored the relevant literature and have developed a model of teachers’ perceptions of school-related factors in special education that proved to be valid.

## 2 Theoretical background

### 2.1 Interpretations of SEN

The history of educating disabled children has followed a relatively similar path in every western society (Bryer and Beamish, 2019). The Salamanca Conference in 1994 was a significant breakthrough with its statement on the education of disabled students, which was agreed at a UNESCO World Conference on Special Needs Education (UNESCO, 1994). It began with a commitment to education for all, recognising the necessity and urgency of providing education for all students – both young people and adults alike – within the regular education system. The statement declared that “schools should accommodate all children regardless of their physical, intellectual, social, emotional, linguistic or other conditions. This should include disabled and gifted children, street and working children, children from remote or nomadic populations, children from linguistic, ethnic or cultural minorities and children from other disadvantaged or marginalised areas or groups” (The Salamanca Statement and Framework for Action on Special Needs Education, para 3, United Nations Educational, 1994). In 2000, the principles of inclusive education were re-stated at the Dakar World Education Forum (The Dakar Framework for Action, 2000). Participants adopted a framework committed to achieving quality education for all students, which was confirmed by world leaders in the Millennium Declaration (Bhaskara, 2003). A few years later in Malaga, the Council of Europe (2003) declared that education is the essential tool to enable disabled children to integrate into society; it established a legislative framework for the inclusion of SEN students in mainstream schools (Council of Europe, 2003). The Action Plan, 2006–2015 (Council of Europe, 2006), focused on SEN students' needs by supporting and promoting the facilitation of their education (Council of Europe, 2006). This may be challenging, since different countries apply the same educational concept in different ways. For example, ‘inclusion’ is a complex and misused concept (see Mitchell, 2005). Inclusion highlights children's right to receive education together with their peers; therefore, each school needs to be prepared to welcome everyone with or without disabilities. Developing students' abilities without labelling is the fundamental principle of inclusion. Due to this conception, it is commonly known as a “pedagogy of diversity” or “pedagogy for everyone” (Krawitz, 1995).

The term *special educational needs* has also been addressed by several significant international statements. It was first referred to in the Framework of the Salamanca World Conference as “all those children and youth whose needs arise from disabilities or learning difficulties” (The Salamanca Statement and Framework for Action on Special Needs Education, para 3, United Nations Educational, 1994). Ireland's Education for Persons with Special Educational Needs Act (2004) defined it as “a restriction in the capacity of the person to participate in and benefit from education on account of an enduring physical, sensory, mental health or learning disability or any other condition which results in a person learning differently from a person without that condition” (para 1). However, as definitions of SEN are specific to each country's legislation, they differ widely across countries. Based on the international and national literature, inclusive education has a decisive impact on SEN and on developing learners (Goncalves and Lemos, 2014). Including students with SEN in mainstream schools is an international development which is supported by national legislation and given expression in statements and reports issued by prominent international bodies, such as the United Nations and the Council of Europe.

### 2.2 The role of ICT in the development of SEN students

Nowadays, the use of information and communication technologies (ICT) has become an integral part of our daily lives; therefore, it is essential to integrate them into instructional practices (Çapuk, 2015; Palomino, 2017). ICT devices and ICT-related teaching methods can positively affect students’ achievement in ICT use and communication (Comi et al., 2017). Technology-based teaching and learning can provide a number of exceptional opportunities in the various areas of the teaching and learning process. It can play an important role in enhancing knowledge acquisition and in the development of specific skills. Technology-based tools and equipment can facilitate the acquisition of almost all subjects (Baglama et al., 2017; Ozdamli, 2017). In addition, ICT offers complementary support tools for teachers; for example, in measurement and testing processes, technology-based assessment can provide instant feedback that shortens working time and improves efficiency (Csapó and Molnár, 2019; Molnár and Csapó, 2019).

A further advantage is that it can be applied as a personal support tool to enable SEN students to achieve greater independence in learning and thus foster greater participation in the mainstream environment. ICT devices that facilitate simultaneous use of different sensory channels can aid in enhancing learning for these students. When a variety of visual, auditory and kinaesthetic ICT-based learning tools are employed, the learning process can be adapted to students’ preferred channel of stimulus reception (Martínez, 2011). Obradović et al. (2015) found that creative teaching and a multisensory approach with ICT tools have a rather positive effect on students with learning disabilities. Moreover, they provide opportunities for every student to be more active and to excel because of their motivating power (Delić-Zimić and Gadžo, 2018).

However, Comi et al. (2017) results suggest that the effectiveness of ICT in education strongly depends on the actual tasks and on the teachers' ability to integrate ICT methods into their teaching process. ICT tools should be used in a purposeful way to provide opportunities for all students to learn better and faster in an enjoyable environment (Çapuk, 2015). Therefore, it is essential that teachers be prepared with the necessary knowledge and skills during teacher training and in their teaching practice (Delić-Zimić and Gadžo, 2018).

### 2.3 The context of the study

In Hungary there is a growing trend in the number of SEN students. From the 2012–13 academic year, the Act on Public Education revised the definition of special educational needs. According to the new definition, SEN refers to “the deviating development caused by disorders relating to intellect, vision, hearing, physical (locomotor) and speech organs or other psychological development disorders (severe and permanent disorders of cognitive functions or behavioural development)”.^1^

Neither the previous nor the new Hungarian Act on Public Education sets down any provisions for students with SEN being educated in special institutions. It allows both options, and parents enjoy the right to select an inclusive educational institution, where SEN students receive special and guided development on the basis of the relevant committee's expert opinion. In line with international practices, inclusive education has also come to the fore in Hungary. Almost the entire educational sector there supports forms of inclusive education. In the last school year, there were more than 88,000 SEN students in the education system. There were more than 9,100 SEN children in kindergartens, more than 55,000 students with SEN in primary schools, and more than 23,000 SEN pupils in secondary education (KSH, 2017–18). According to European Agency data, more than half of SEN students, 60%, were educated in an inclusive environment in Hungary. The accuracy of this data is questionable since the categorisation does not always match practice and since certain elements depend on legal regulations, for example, the definition of SEN. Therefore, according to the data cited above, most Hungarian SEN students are taught in inclusive classrooms, which should rather be considered a type of inclusive education (because of the mismatch between theory and practice). Typically, these children have some kind of mild disability, most commonly some type of learning disability. Another problem is the actual form of inclusive education. Although we can find different levels of inclusion in practice, ranging from rigid to full, we have no information on schools' documentation on the different types.

It is a fact that Hungarian teachers have inadequate knowledge of SEN as a result of the curriculum at universities, where courses on this topic are only optional. However, teachers' attitudes towards SEN and inclusive education have received increasing attention during the last few years, emphasising an improvement in study results and a more positive school experience for students with disabilities (Sharma et al., 2018). At universities where there these issues are stressed, pre-service teachers receive intensive and in-depth training on effective teaching methods for SEN students. These courses include the latest interpretations of SEN students’ development supplemented by ICT methods, such as watching videos, films and audio materials, and searching for e-learning materials and using developmental programmes (Sharma et al., 2018).

Despite the fact that teachers' perceptions of SEN and inclusive education have taken centre stage during the last few years, not every Hungarian teacher acquires adequate knowledge of SEN. In addition, we know little about teachers’ perceptions of certain SEN-related factors. With regard to ICT use in Hungarian schools, the technical infrastructure in schools has been highlighted in research (Molnár and Csapó, 2019; Vidákovich and Habók, 2019), but we do not have a comprehensive picture of teaching practices and use of ICT in developing SEN students.

The Department of Education and Science in Dublin have developed guidelines (Inclusion of Students with Special Educational Needs Post Primary Guidelines) for schools that can assist in the provision of appropriate education for SEN students (Stack, 2007). These guidelines present a whole-school approach to assist management and staff in elaborating a policy framework to provide adequate education for SEN students. The guidelines also offer recommendations on how existing resources can be involved in everyday work. The main issues in the guidelines deal with the following school-related factors: the quality of school planning, the quality of professional development, the quality of curriculum provision and the quality of learning and teaching.

## 3 Research questions

**Figure 1:**
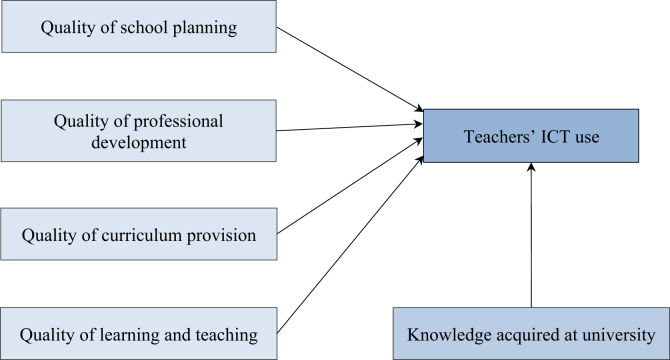
Hypothesized model of teachers' perceptions of school-related factors in special education.

We have developed a hypothesized model of teachers' perceptions of school-related factors in special education (Figure 1) based on the relevant literature and the Irish guidelines. In the model, we regarded the central role of ICT and digital tools in their education, such as searching for and employing different kinds of digital materials in the learning process. Teacher training courses can also be decisive factors for teachers using digital materials with SEN students. As Figure 1 shows, our research hypothesizes strong relations between the variables and assumes that both the fields suggested by the guidelines and teachers' knowledge have significant effects on teachers’ ICT use.

The main objective of our study was to test this model and investigate teachers’ perceptions of SEN-related factors and to map the relations between the variables. We aimed to answer the following research questions:

RQ1: What are teachers’ perceptions of school-related factors as regards special education in their school?

RQ2: To what extent are ICT tools involved in teachers’ teaching practice as regards SEN education?

RQ3: What relationships can be perceived between the school-related factors of teaching- and learning-related issues, and how do these factors and teachers' knowledge acquired at university affect teachers’ ICT use?

## 4 Methods

### 4.1 Participants

The data was collected from 121 teachers (N_male_ = 20; N_female_ = 101). All of them teach in schools attended by SEN students. 110 teachers teach SEN students both in class and in extracurricular activities; however, eleven teachers only meet SEN students during extracurricular activities and school events. With regard to teachers' qualifications, one part of our sample was only qualified to teach students with special educational needs and disabilities. The other part of the sample had completed SEN-related courses during their Master's studies or other postgraduate or doctoral studies. We did not specify the exact level of qualifications on the basis of the course or training in which they participated, but each teacher had previously acquired at least a basic knowledge of SEN. 116 teachers reported that students struggle with multiple difficulties in their school, 13 teachers reported that their school educates students with learning disabilities, one teacher noted children with mental disabilities, and one teacher described other disabilities which were not specified. Thirty-six teachers had 11–20 years of work experience, and five teachers had 5–10 years teaching practice. Thirteen teachers had been on the job for four or fewer than four years. On the whole, our respondents were experienced, with more than half of them reporting 20 or over 20 years of work experience. Accordingly, more than half of the participants are between 41 and 60 years of age. In Hungary, primary school lasts for eight years (Years 1–8), so we had 76 teachers who teach at this level. There were also 45 teachers who work in secondary school (Years 9–12) (Table 1). The Kolmogorov–Smirnov test indicated that our sample is not representative of the entire population of Hungarian teachers (D (121) = 0.228, p = 0.00).

**Table 1:** The details of the sample.

Age group	Number of teachers (male/female)	Number of primary/secondary school teachers	Years of teaching practice				
			1–2 years	3–4 years	5–10 years	11–20 years	over 20 years
20–30	7 (3/4)	6/1	2	5			
31–40	14 (2/12)	10/4		2	4	8	
41–50	47 (9/38)	28/19	2	2		22	21
51–60	48 (1/47)	32/16				5	43
61–70	5 (5/-)	-/5			1	1	3
Total	121 (20/101)	76/45	4	9	5	36	67

### 4.2 Instruments

To explore teachers' perceptions of their teaching-related practices, we adapted the *Audit of provision for students with special educational needs (Audit)* self-evaluation scale from the ‘Inclusion of Students with Special Educational Needs Post-Primary Guidelines’ (Stack, 2007, see Appendix 1). The reason why we have chosen this questionnaire is that it covered all of our main research objectives and it was previously validated for 140 teachers in Hungary. An exploratory factor analysis (EFA) was conducted to reinforce the factor structure of the questionnaire. The analysis indicated a meritorious KMO index (.884), and Bartlett's test (Chi-square = 1419.703; df = 300, p < .001) also reinforced the unidimensionality of the scale. However, CFA could not verify the suspected relationships that were established in the *Audit*, although the distribution of the subscales was similar in our research. While the *Audit* involved four distinct factors [quality of school planning (ten items), quality of professional development (three items), quality of curriculum provision (four items) and quality of learning and teaching (eight items)], the analysis showed seven distinct categories (Chi-square = 381.459; df = 254; p < .001; CFI = .912; TLI = .888; RMSEA = .058). These categories involved quality of school planning – general (three items: items 1, 2 and 4), e.g. ‘The school's admissions policy facilitates the admission and participation of students with special educational needs'; quality of school planning – staff (three items: items 3, 5 and 6), e.g. ‘The school liaises with relevant external agencies regarding provision for students with special educational needs’; quality of school planning – documents (four items: items 7–10), e.g. ‘The school plan includes references to realistic and practical provision for students with special educational needs’; quality of professional development (three items: items 11–13), e.g. ‘All staff are encouraged and facilitated to participate in appropriate professional development in special education’; quality of curriculum provision (four items: items 14–17), e.g. ‘Co-curricular and extracurricular activities that support and enhance learning are open and accessible to students with special educational needs’; quality of learning and teaching – planning (five items: items 18–22), e.g. ‘There is a direct link between the school plan and the learning and teaching programmes provided for students with special educational needs’; and quality of learning and teaching – teachers' competence (three items: items 23–25), e.g. ‘Modes of assessment and feedback (including homework) are differentiated by teachers in relation to their students’ ability’.

Since *Audit* did not involve statements on teachers' ICT usage, we have added an ICT section to it and developed the Questionnaire for SEN Teachers (QST) on the basis of this revised scale for the *Audit*. It consisted of three parts. In the first part, we asked about background data, such as information on participants' qualifications and teaching experience and on the rate of SEN students and specialists or specially trained staff in the schools. The second part comprised the seven subfields of the *Audit* scale. Teachers responded on a four-point scale to the statements, consisting of ‘under consideration’, ‘being developed’, ‘established’ and ‘regularly being reviewed and improved’. The third part examined teachers' ICT usage with and without SEN students. We developed the items on this subscale ourselves based on our previous experience, e.g. ‘How often do you search for e-learning materials designed for SEN students?’ The responses on the four-point Likert scale ranged from ‘almost never’ to ‘almost always’.

**Figure 2:**
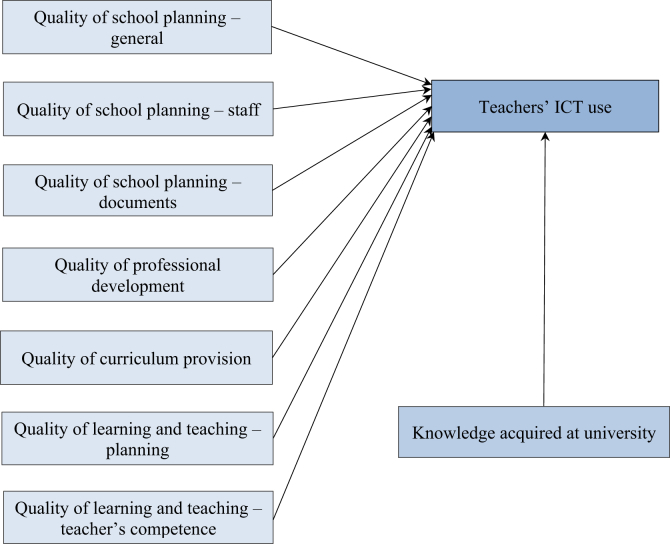
Revised hypothesized model of teachers' perceptions of school-related factors in special education.

Based on these findings, we reconsidered our first model (Figure 1) and improved it as shown in Figure 2. In our revised hypothesized model, we applied the seven distinct factors in the *Audit* and assumed that all these factors and teachers’ knowledge have a significant impact on ICT use among teachers.

### 4.3 Design and procedure

In the research quantitative methodology has been used. Since our questionnaire was designed for educators teaching SEN students, we sent our online measurement tool to institutions where we knew SEN students were taught. First, we contacted the school management, who decided whether to forward the link to the questionnaire to the teaching staff. After that, teachers decided whether to complete the measurement tool. Participation was voluntary, and full anonymity was guaranteed. No personal data, name or e-mail address was stored. The research was carried out with the ethical approval of the IRB at the Doctoral School of Education, University of Szeged and informed consent was obtained from the participants. The respondents completed the measurement tool individually at a freely chosen time. After logging onto the questionnaire website, participants read research information and confirmed their participation in the research. We only involved responses which were released for data analysis. Participants first provided background data related to their age, qualification, school, etc. Second, respondents completed the QST questionnaire. Completing the entire measurement tool took approx. 15–20 min.

Data were processed and analysed using descriptive statistics. We also examined the internal consistency of the questionnaire at the subscale level. Correlation coefficients between variables were calculated to ascertain whether a path analysis could be conducted. The path analysis attempts to indicate relationships between observed and latent variables. In our research, we aimed to discover possible paths between school- and learning-related factors and ICT usage variables. The Comparative Fit Index (CFI), Normed Fit Index (NFI) and root mean square error of approximation (RMSEA) were used as fit indices to verify our model. We used IBM Statistics SPSS and AMOS 23.0 for the data analysis.

## 5 Results

### 5.1 Descriptive analysis

**Figure 3:**
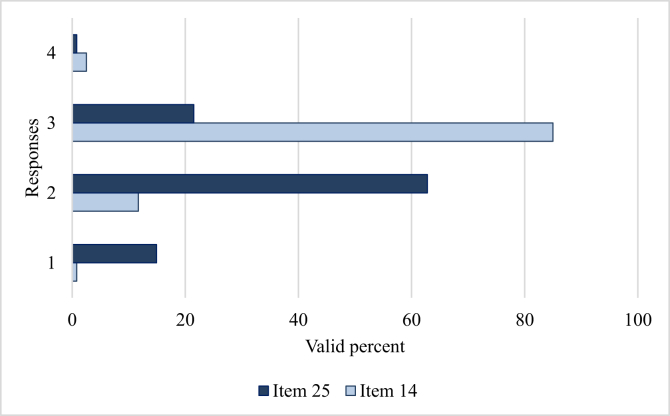
The teachers' responses with the highest and lowest ratings (Items 14 and 25).

We examined the internal consistency (Cronbach's α), means and standard deviation of the QST at the factor levels. Reliability analysis showed that the QST has an acceptable reliability between .63 and .77. Table 2 shows the result of the descriptive analysis. Overall, most teachers stated that inclusive education in their school is in the “being developed” or “established” phase. Teachers considered the quality of curriculum provision as the most important factor in special education (M = 2.65; SD = .41, p < 001). They rated the following statements the highest: ‘The students with special educational needs have access to certificate and school-based programmes which are appropriate to their needs and interests’ (M = 2.89; SD = 0.41) and ‘The students with special educational needs have access to a range of subjects at varying levels appropriate to their needs and interests’ (M = 2.81; SD = .52). The following statement was rated the lowest: ‘There are procedures to monitor the actual achievement of students with special educational needs to ensure it is in keeping with their ability and in accordance with their individual plan’ (M = 2.08; SD = .63) (Figure 3).

**Table 2:** Teachers’ perceptions of school-related factors in special education.

Factors	Crb α	M	SD	Skewness		Kurtosis	
				Stat.	SE	Stat.	SE
Quality of school planning – general	.70	2.39	.50	-.58	.22	.16	.44
Quality of school planning – staff	.63	2.57	.46	-.70	.22	.36	.44
Quality of school planning – documents	.72	2.41	.52	-.47	.22	-.40	.44
Quality of professional development	.63	2.37	.54	-.66	.22	-.03	.44
Quality of curriculum provision	.71	2.65	.41	-.19	.22	1.82	.44
Quality of learning and teaching – planning	.77	2.38	.47	-.46	.22	.25	.44
Quality of learning and teaching – teachers' competence	.72	2.20	.48	.02	.22	.69	.44
ICT use	.77	2.51	.58	-.26	.22	.48	.44

As regards teacher's ICT use, teachers rated the use of e-diaries and preparing presentations the highest (M = 3.33; SD = 1.15; p < .001), while videos, films and audio materials were used with significant frequency (M = 3.00; SD = .84). They rated searching for e-learning materials for all students somewhat lower (M = 2.66; SD = .84). Developmental programmes for all students were also reported to be employed relatively often, which is very important in teaching (M = 2.26; SD = .87). Using e-libraries was not particularly common (M = 1.98; SD = .93). Items related to SEN students were rated relatively low, that is, searching for e-learning materials designed for SEN students (M = 2.29; SD = .83) and using developmental programmes designed for SEN students (M = 1.97; SD = .87).

### 5.2 Multivariate analyses

**Figure 4:**
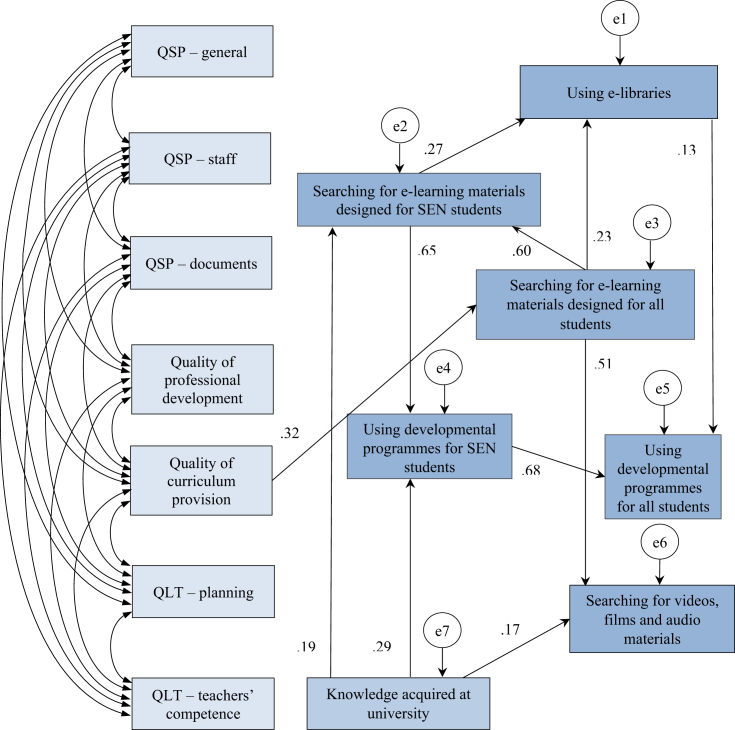
Final model of teachers' perceptions of school-related factors in special education. QSP – general: Quality of school planning – general; QSP – staff: Quality of school planning – staff; QSP – documents: Quality of school planning – documents.

We tested our hypothesized model to discover the possible relationships between the QST factors, that is, the factors of teaching- and learning-related issues, teachers' knowledge acquired at university and their ICT usage. As shown in Figure 2, we involved the following factors in the revised *Audit*: quality of school planning – general; quality of school planning – staff; quality of school planning – documents; quality of professional development; quality of curriculum provision; quality of learning and teaching – planning; and quality of learning and teaching – teachers' competence. Teachers' ICT use was mapped by eight statements: searching for e-learning materials designed for SEN students; searching for e-learning materials designed for all students; using developmental programmes designed for SEN students; using developmental programmes designed for all students; searching for videos, films and audio materials; using e-libraries; using e-diaries and preparing presentations. Two of these components did not fit the data; therefore, we eliminated them (using e-diaries and preparing presentations) from the final version of our model (Figure 4). We also asked about teachers’ knowledge acquired at university.

Correlation analysis was conducted to analyse the relations between the categories and variables (Appendix 2). The result showed significant correlational coefficients between the learning and teaching related factors (r = .22–.67, p < .05–.01). Significant correlation coefficients were also found among the ICT usage of the teachers (r = .19–.71, p < .05–.01). Teachers’ knowledge acquired at university also showed a significant correlation with almost all the ICT items: searching for e-learning materials designed for SEN students (r = .33, p < .01), using developmental programmes for SEN students (r = .40, p < .01), using developmental programmes for all students (r = .29, p < .01), preparing presentations (r = .20, p < .05), and using videos, films and audio materials (r = .21, p < .05).

We also ran a path analysis to test the relations and directions between the variables and to identify direct and indirect connections between them. Data indicated a non-fitted model (Chi-square = 418.865; df = .28; p = .000; CFI = .030; TLI = -.558; NFI = .065; RMSEA = .341); therefore, we modified our assumptions and attempted to discover the possible paths. Originally, we regarded ICT use as one factor; however, to obtain a more detailed picture of the relations between the variables for the ICT factor, we considered them as individual components. Finally, we managed to build a well-fitting model that we present in Figure 3 (Chi-square = 80.408; df = .59; p = .033; CFI = .971; TLI = .948; NFI = .904; RMSEA = .055). A one-headed arrow shows possible paths between variables, while two-headed arrows indicate correlations.

With regard to school-related factors in the model we constructed, we observed significant correlational coefficients between learning- and teaching-related factors (r = .30–.67) and all relationships are significant (p < .05). The only direct effect of teaching- and learning-related issues on ICT usage can be observed between quality of curriculum provision and searching for e-learning materials designed for all students (ß = .32). The strongest effect of using developmental programmes designed for SEN students can be found on using developmental programmes designed for all students (ß = .68). The searching for e-learning materials designed for SEN students factor also has a considerable effect on using developmental programmes designed for SEN students (ß = .65). The direct effect of searching for e-learning materials designed for all students is strong on searching for e-learning materials designed for SEN students (ß = .60). The searching for e-learning materials designed for all student's factor significantly influences the frequency of searching for videos, films and other audio materials (ß = .51). Knowledge acquired at university has a notable direct effect on using developmental programmes designed for SEN students (ß = .29) and a smaller but significant effect on searching for e-learning materials designed for SEN students (ß = .19) and on searching for videos, films or other audio materials (ß = .17). Using e-libraries is influenced by searching for e-learning materials designed for SEN students (ß = .27) and for all students (ß = .23) to a lesser degree. A lower but significant effect can be observed in using e-libraries on using developmental materials for students (ß = .13).

We also analysed the indirect effect of quality of curriculum provision of ICT usage variables. We discovered the indirect effects of quality of curriculum provision of ICT usage on searching for e-learning materials designed for SEN students through searching for e-learning materials designed for all students (ß = .19). On the same path, quality of curriculum provision influenced searching for audio materials (ß = .16) and e-library usage (ß = .12). Using developmental programmes designed for SEN students (ß = .13) and, to a lesser extent, for all students (ß = .10) is determined by quality of curriculum provision through two variables.

## 6 Discussion

The main objective of our research was to explore teachers' perceptions of special educational needs (SEN) in Hungary and to map their teaching practices in relation to ICT tools involved in classes with both SEN and typically developing students. We built a hypothesized model on the basis of the relevant literature and tested it to explore the connections and effects of variables. The structure of the model was based on the Inclusion of Students with Special Educational Needs Post Primary Guidelines written by the Department of Education and Science in Dublin (Stack, 2007), supplemented with the crucial role of ICT and digital tools and knowledge that teachers acquired at university during their teacher training courses. We assumed that both the fields suggested by the guidelines and teachers’ knowledge acquired at university have significant effects on digital methods used by teachers.

As regards teachers' perceptions of school-related factors in special education, our results showed that teachers rated the role of quality of curriculum provision the highest. They reported that SEN children have access to a range of subjects at varying levels appropriate to their needs and interests. They also gave a high rating to the statement that their SEN students can participate in school events, extracurricular activities and developmental sessions at the institutional level. Materials required for the teaching of SEN students were reported to be available for school staff, and SEN students were said to be able to obtain certificates. However, as Hornby (2015) argue, the main point would be not merely to involve SEN students in mainstream classes, but to design special curricula for children with SEN, which are more appropriate for them with requirements adjusted to their needs. Most other European studies reported such trends (Chitiyo and Brinda, 2018; Schwab et al., 2017; Seçer, 2010). However, the lowest means were recorded in teachers' competences. The statements indicated that teachers reported difficulties in using a wide variety of teaching strategies and adapting them to students' skills and needs. They also stated that differentiated teaching methods are not widespread. In addition, very few teachers considered that they can monitor the current development of their SEN students and use adaptive teaching methods. This can be explained by the fact that not all teacher trainees acquire comprehensive knowledge in their university education courses (Chitiyo and Brinda, 2018). In teacher training, students only have a few courses in SEN; hence, teachers must broaden their knowledge in postgraduate courses or prepare individually for the various special needs. Therefore, the use of adaptive techniques and evaluation methods requires the most preparation for teachers' teaching practice. Our results are in line with other Hungarian studies that have also pointed out the responsibility of teacher education in this area (Sharma et al., 2018). Seçer’s (2010) findings have also confirmed the value of professional development as a key to successful inclusiveness. Their results have shown that in spite of the growing number of SEN students in Turkey, the majority of teachers cannot use adequate teaching techniques in the inclusive classroom. Guerra et al. (2017) findings have also reinforced the fact that the majority of teachers have not been adequately trained to teach students with ADHD. Clearly, this would be essential. However, there is a positive trend in this regard in some European countries, such as in Spain, where courses on special education are offered to teacher trainees to prepare them with adaptive teaching methods (Lasagabaster and de Zarobe, 2010).

We also examined to what extent ICT tools are involved in teachers’ teaching practice in connection with SEN education. Our findings demonstrated that teachers make moderate use of ICT tools in their teaching processes and that they moderately seek out and use different types of ICT materials not only for the majority of students, but also for SEN students. Teachers considered the use of e-diaries, preparing presentations and using videos, films and other audio materials the most often. They reported lower use of searching for e-learning materials and using developmental programmes for all students. These items for SEN students were reported even lower. However, a number of research reinforced the significance of ICT tools in developing SEN students (Martínez, 2011; Ozdamli, 2017). Numerous studies have also highlighted the crucial role of ICT tools in higher education (Nagy and Habók, 2018; Palomino, 2017). If trainees used ICT tools more actively during their university studies, they would be more likely to apply ICT methods frequently in their teaching practice (Baglama et al., 2017; Yeni and Gecu-Parmaksiz, 2016). The least emphasised area was using e-libraries, although e-libraries are the most important sources for teachers to find articles, books and methodological materials. However, such libraries are mainly in foreign languages, mostly English, and we cannot be certain that teachers have the adequate language proficiency to be able to work with these tools and materials.

We also tested our hypothesized model and investigated what relationships can be perceived between school-related factors of teaching- and learning-related issues and how these factors and teachers' knowledge acquired at university affect teachers' ICT use. After reconsidering and modifying the original four-factor structure of the adapted scale for the *Audit* and applying seven distinct factors, we assumed that all these factors with teachers' knowledge have a significant impact on teachers' ICT use. In the final model, we involved the seven learning- and teaching-related factors, teachers' knowledge acquired at universities and teachers' ICT use. As teachers’ ICT use was central to our investigation, we regarded the variables as individual components. Two of the statements did not fit the data; therefore, we eliminated them (using e-diaries and preparing presentations). A path analysis showed significant connections between learning- and teaching-related factors. We also observed significant relationships between ICT variables, but the effects between the learning- and teaching-related and ICT factors proved to be rather limited. The quality of curriculum provision turned out to be the only significant factor which has an essential effect on using ICT teaching materials. This may stem from the fact that differentiated methods for SEN students and ICT use are not closely connected in the teaching practice of the sample. We also found a moderate and limited use of ICT materials in the sample. Participating teachers mostly search for videos, films and audio materials that can be located quickly and can easily be inserted into the learning materials and school classes. This also indicates that the general ICT use in our sample is characterised by ready-made materials, not by teacher-made ones. Moreover, teachers primarily search for e-learning materials for all students, not specifically for SEN students, although the latter would be a more adaptive method to support the various visual, auditory or kinaesthetic sensory channels of their SEN students (Comi et al., 2017; Yeni and Gecu-Parmaksiz, 2016). University SEN courses can greatly aid teachers in preparing them to search for and employ digital materials and developmental programmes in teaching SEN students. Moreover, teachers who are also highly proficient ICT users can use ICT tools for SEN students more effectively.

## 7 Conclusions, limitations and pedagogical implications

To recap briefly, this study demonstrates that teaching SEN students in an integrated education system poses unique challenges; it is therefore a relevant topic and an issue to be addressed. Teachers should be encouraged to develop and use new exercises, good practices and innovative solutions. As our hypothesized model of teachers' perceptions model indicates, there is no direct link between teachers’ perceptions of school-related factors of teaching- and learning-related issues and their ICT use as regards SEN students. Teachers in mainstream schools typically do not possess high-level skills to deal with SEN students, and they do not possess high-level competences in using ICT in teaching practice.

The primary significance of the study is that it draws attention to the importance of SEN courses at universities and the significance of certain school-related factors that can assist management and staff in elaborating a policy framework to provide adequate education for SEN students.

However, our research has some limitations. First of all, the questionnaire contains no items on personal and material conditions. Although we are aware of their importance, our focus in this research was more on the operation of schools and the principles underlying it. In the future, we plan to investigate the issue of material conditions as well. Monsen et al. (2014) expanded a previous New Zealand study on the impact of teachers' attitudes towards inclusion in classroom learning environments and found that teachers’ attitudes towards inclusion were significantly influenced by support for their work (the existence of appropriate personal and material conditions). The second limitation is the number of participants. As regards the age and proficiency distribution of the sample, it was not representative of the population of Hungarian teachers.

The questionnaire also focused on the quality of teaching, which more than half of the sample considered to be improving. As noted above, it is necessary to work together in one team consisting of students, teachers, parents and special educators. Our research findings are of potential value both domestically and internationally as well, since an increasing number of SEN children are encountered at all levels of education.

## Declarations

### Author contribution statement

A. Habók: Conceived and designed the experiments; Performed the experiment; Analyzed and interpreted the data; Contributed reagents, materials, analysis tools or data; Wrote the paper.

A. Krausz: Conceived and designed the experiments; Performed the experiment; Contributed reagents, materials, analysis tools or data; Wrote the paper.

I. D. Kapás: Conceived and designed the experiments; Wrote the paper.

A. Magyar: Analyzed and interpreted the data; Contributed reagents, materials, analysis tools or data; Wrote the paper.

### Funding statement

This work was supported by the University of Szeged Open Access Fund, Grant number: 4641. Anita Habók was supported by the János Bolyai Research Scholarship of the Hungarian Academy of Sciences.

### Competing interest statement

The authors declare no conflict of interest.

### Additional information

No additional information is available for this paper.

## Figures and Tables

**Table:** 

Statements	M	SD
1. The school's mission statement reflects an inclusive ethos.	2.33	.58
2. The school's admissions policy facilitates the admission and participation of students with special educational needs.	2.53	.65
3. The school engages actively with relevant primary school personnel regarding provision for students with special educational needs.	2.69	.63
4. The school adopts a whole-school and systematic approach to identifying, providing for and reviewing the educational requirements of students with special educational needs.	2.32	.67
5. The school actively promotes parental involvement and facilitates contact between parents and teachers.	2.46	.63
6. The school liaises with relevant external agencies regarding provision for students with special educational needs.	2.56	.58
7. The school plan includes references to realistic and practical provision for students with special educational needs.	2.35	.78
8. The school's discipline policy/code of behaviour is suitably flexible to take account of individual differences.	2.32	.73
9. The school's homework policy includes reference to students with special educational needs.	2.29	.76
10. The school's assessment policy includes reference to students with special educational needs including reasonable accommodations for certificate examinations.	2.71	.56
11. All staff are encouraged and facilitated to participate in appropriate professional development in special education.	2.22	.68
12. All staff are fully aware of their roles and responsibilities regarding students with special educational needs.	2.43	.68
13. A named teacher has been designated as the teacher with responsibility for coordinating and liaising with colleagues and other relevant professionals in relation to students with special educational needs.	2.45	.75
14. The students with special educational needs have access to certificate and schoolbased programmes which are appropriate to their needs and interests.	2.89	.41
15. The students with special educational needs have access to a range of subjects at varying levels appropriate to their needs and interests.	2.81	.52
16. Curriculum documents and materials related to provision for students with special educational needs are readily available to staff.	2.32	.72
17. Co-curricular and extracurricular activities that support and enhance learning are open and accessible to students with special educational needs.	2.60	.56
18. There is a direct link between the school plan and the learning and teaching programmes provided for students with special educational needs.	2.38	.71
19. Assessment outcomes are appropriately shared and used to inform learning and teaching.	2.62	.60
20. The school collaboratively creates, implements and reviews educational plans for students with special educational needs.	2.29	.67
21. The school offers an appropriate, safe and stimulating environment for all students creating a sense of belonging and security.	2.49	.65
22. All students are challenged and motivated by teaching and learning activities that are appropriate by level and pace.	2.13	.63
23. All teachers use a variety of teaching strategies and methodologies that take account of the range of student abilities, needs and interests.	2.26	.58
24. Modes of assessment and feedback (including homework) are differentiated by teachers in relation to their students' ability.	2.27	.59
25. There are procedures to monitor the actual achievement of students with special educational needs to ensure it is in keeping with their ability and in accordance with their individual plan.	2.08	.63

**Table:** 

How often do you …	M	SD
1. … search for e-learning materials designed for SEN students?	2.29	0.83
2. … search for e-learning materials designed for all students?	2.66	0.84
3. … use developmental programmes designed for SEN students?	1.97	0.80
4. … use developmental programmes designed for all students?	2.26	0.87
5. … use e-libraries?	1.98	0.93
6. … use e-diaries?	3.33	1.15
7. … prepare presentations?	2.63	1.00
8. … search for videos, films and audio materials?	3.00	0.84

**Table:** 

Questionnaire fields	1	2	3	4	5	6	7	8	9	10	11	12	13	14	15	16
1. Quality of school planning – general	1.00															
2. Quality of school planning – staff	.60	1.00														
3. Quality of school planning – documents	.62	.59	1.00													
4. Quality of professional development	.67	.64	.62	1.00												
5. Quality of curriculum provision	.40	.38	.46	.47	1.00											
6. Quality of learning and teaching – planning	.48	.44	.57	.63	.49	1.00										
7. Quality of learning and teaching – teachers’ competence	.31	.35	.52	.47	.48	.60	1.00									
8. Searching for e-learning materials designed for SEN students	.25	.28	.28	.27	.32	n.s.	n.s.	1.00								
9. Searching for e-learning materials designed for all students	.23∗	.26	.23∗	.22∗	.32	n.s.	n.s.	.62	1.00							
10. Using developmental programmes for SEN students	n.s.	n.s.	n.s.	n.s.	.24	n.s.	n.s.	.71	.50	1.00						
11. Using developmental programmes for all students	n.s.	n.s.	n.s.	n.s.	n.s.	n.s.	n.s.	.60	.62	.72	1.00					
12. Using e-libraries	.18∗	n.s.	n.s.	n.s.	.18∗	n.s.	n.s.	.42	.40	.33	.36	1.00				
13. Using e-diaries	n.s.	n.s.	n.s.	.24	n.s.	.32	n.s.	n.s.	n.s.	n.s.	n.s.	n.s.	1.00			
14. Making presentations	n.s.	n.s.	n.s.	n.s.	n.s.	n.s.	n.s.	.28	.42	.19∗	.32	.33	.28	1.00		
15. Searching for videos, films and audio materials	n.s.	n.s.	n.s.	n.s.	.26	n.s.	n.s.	.37	.52	.25	.31	.25	n.s.	.51	1.00	
16. Knowledge acquired at university	n.s.	n.s.	n.s.	n.s.	n.s.	n.s.	n.s.	.33	n.s.	.40	.29	n.s.	n.s.	.20∗	.21∗	1.00

Correlational coefficients are significant at p<.01; ∗p<.05 level, n.s.: no significance

## Appendix 1 Questionnaire for SEN teachers (QST)

Background data

Gender: Male/Female

Age group: 20–30/31–40/41–50/51–60/61–70

Qualification: BA/MA/Other

Did you have special courses on SEN during your university education? None Many

Teaching experience: 1–2 years/3–4 years/5–10 years/11–20 years/Above 20

Are there SEN students in your institution? Yes/No

If yes, what type of SEN students?

Visual impairment

Visual processing disorder

Hearing impairment

Learning difficulties

Intellectual disability

Physical disability

ADHD

Autistic spectrum disorder

Other

Are there any specialists or specially trained staff in your school?

ICT statements

## Footnotes

1 https://www.european-agency.org/country-information/hungary/legislation-and-policy.
